# NEUROCORRELATES BETWEEN THEORY-OF-MIND AND BILINGUALISM IN GRAY MATTER VOLUME OF YOUNG AND OLDER ADULTS

**DOI:** 10.1093/geroni/igac059.2216

**Published:** 2022-12-20

**Authors:** W Quin Yow, Xiaoqian Li, Kwun Kei Ng, Janice Jue Xin Koi, Juan Helen Zhou

**Affiliations:** Singapore University of Technology & Design, Singapore, Singapore; Singapore University of Technology & Design, Singapore, Singapore; National University of Singapore, Singapore, Singapore; National University of Singapore, Singapore, Singapore; National University of Singapore, Singapore, Singapore

## Abstract

The experience of being bilingual may accrue cognitive reserve against age-related declines in older adults. Early bilingualism, e.g., second language age-of-acquisition (L2AoA), has been shown to be associated with better theory-of-mind (ToM) performance in older adults (Yow et al., 2021). Here, we aim to understand the brain structural correlates associated with bilingualism and ToM performance in normal aging. Forty-six young (YA, aged 19-30, M=21.87) and 51 older adult bilinguals (OA, aged 54-77, M=63.61) completed 1) ToM assessments, where they viewed vignettes and answered questions about the protagonists’ mental states, 2) an anatomical MRI scan, 3) a demographic questionnaire including L2AoA and years of education, and 4) a general cognitive ability assessment by the Montreal Cognitive Assessment (MoCA) test. As expected, ANCOVA on ToM composite scores revealed a significant main effect of age – YA showed better ToM performance than OA, F(1,93)=9.48, p=.003, controlling for education and MoCA scores. Importantly, MRI data were preprocessed to obtain gray matter volume (GMV), proxy of neuronal density, of 430 brain regions. Partial least square correlation analysis identified one significant multivariate pattern linking individual differences in GMV with ToM score and L2AoA (48.7% covariance explained, p=.014). Regardless of age, larger GMV in several regions including prefrontal, frontal, medial temporal, and superior temporal cortices were associated with earlier L2AoA (p=.003) and higher ToM score (p=.004), indicating shared variance between ToM and L2AoA in brain morphology. Findings suggest that earlier bilingual acquisition might promote brain maturation that would preserve ToM ability well into later stages of life.

